# Synthetic data generation: a privacy-preserving approach to accelerate rare disease research

**DOI:** 10.3389/fdgth.2025.1563991

**Published:** 2025-03-18

**Authors:** Jorge M. Mendes, Aziz Barbar, Marwa Refaie

**Affiliations:** ^1^ Comprehensive Health Research Centre (CHRC), NOVA Medical School, Faculdade de Ciências Médicas, Universidade NOVA de Lisboa, Lisbon, Portugal; ^2^ Faculty of Engineering and Computer Science, American University of Science and Technology, Beirut, Lebanon; ^3^ School of Computing, Coventry University, The Knowledge Hub Universities, New Administrative Capital, Egypt

**Keywords:** synthetic data, medical imaging, European Health Data Space (EHDS), privacy preservation, rare disease research, AI-driven diagnostics, regulatory compliance, ethical frameworks

## Abstract

Rare disease research faces significant challenges due to limited patient data, strict privacy regulations, and the need for diverse datasets to develop accurate AI-driven diagnostics and treatments. Synthetic data—artificially generated datasets that mimic patient data while preserving privacy—offer a promising solution to these issues. This article explores how synthetic data can bridge data gaps, enabling the training of AI models, simulating clinical trials, and facilitating cross-border collaborations in rare disease research. We examine case studies where synthetic data successfully replicated patient characteristics, and supported predictive modelling and ensured compliance with regulations like GDPR and HIPAA. While acknowledging current limitations, we discuss synthetic data’s potential to revolutionise rare disease research by enhancing data availability and privacy file enabling more efficient and effective research efforts in diagnosing, treating, and managing rare diseases globally.

## Introduction

Rare disease research faces critical challenges due to the scarcity of patient data, which stems from small, geographically dispersed populations and fragmented data across institutions (1–4). Privacy regulations such as GDPR and HIPAA restrict access to essential datasets, limiting the potential for AI-driven diagnostics and treatment advancements (5). These barriers often result in underpowered studies, hindering efforts to understand rare diseases and develop targeted therapies comprehensively.

Synthetic data offers a promising solution by generating artificial datasets replicating actual patient data’s statistical properties without containing sensitive information. This approach facilitates data sharing, enhances collaboration among researchers, and ensures compliance with stringent privacy laws (6, 7). By providing diverse and privacy-preserving datasets, synthetic data enables AI models to improve the detection of rare genetic markers and accelerates innovation in diagnostics and treatments.

This perspective article explores the transformative role of synthetic data in overcoming the barriers in rare disease research. The objectives are to define synthetic data and its applications, examine its potential to address challenges in clinical validation and highlight ethical, regulatory, and quality considerations. The paper also proposes best practices for leveraging synthetic data to support equitable and effective healthcare solutions for rare diseases.

## The challenge of limited data in rare disease research

Rare disease research faces significant challenges due to limited data, impeding the understanding of disease mechanisms, therapy development, and diagnosis (8–10).

A lack of expertise and incomplete understanding lead to delayed or incorrect diagnoses and restricted access to multidisciplinary healthcare (11). Limited data hampers clinical trial design, especially with small, heterogeneous patient populations (12).

Poor understanding of rare disease pathophysiologies, limited natural history, and inadequate animal models complicate defining clinical endpoints and data utilisation (13). Small patient populations, low disease awareness, and limited healthcare access hinder dose-finding studies and targeted enrolment. These challenges often lead to reliance on anecdotal evidence rather than robust data (14). The scarcity of patients complicates recruitment for conventional research clinical trials, resulting in underpowered studies and inconclusive results (15). This uncertainty complicates regulatory approval and market access for new therapies and hinders assessment of treatment outcomes and clinical trial endpoints (12, 16).

## The concept of synthetic data

Synthetic data refer to artificially created information that mocks real-world observations. Synthetic data generation becomes crucial when real data is not available due to privacy issues or the rarety of certain observations. Various techniques were proposed for generating synthetic medical data, including tabular, imaging, and omics data. These methods can be classified into rule-based approaches, statistical modelling, and machine learning-based techniques.

1. Rule-based approaches: mimics real world data using predefined rules, constraints and distribution to create artificial data. It can create synthetic patience records based on statistical distribution such as age, gender, etc..
2. Statistical modelling: relies on capturing the relationships between variables in real medical data to generate data with comparable characteristics. Different techniques can be utilised as Gaussian Mixture Model, Bayesian Networks (depending on probabilistic relations between variables) or Markov chains to generate sequence of data (history visit, blood analysis, etc.).
3. Machine learning-based techniques: is considered as a state-of-the-art method such as Virtual Autoencoders (VAEs) or Generative Adversarial Networks (GANs) with different architectures that promote generating different datasets. These datasets can include tabular data, MRI images, radiomic data or bio-signals data like ECG and EGG.
  • GANs are one of the most utilised techniques nowadays to generate artificial data. There are different architecture dealing with the different types of data. In general, GANs operate by training two neural networks, a generator and a discriminator. The generator aims to produce synthetic data that closely resembles real data, while the discriminator tries to classify produced samples as real or synthetic data. These networks are trained together, and through this process, the generator improves its ability to create highly realistic synthetic data over time. GANs evolved into various architectures, each suited for different types of data such as tabular records, MRI, genetic data or even as ECG and ECC signals. For images, deep convolutional GANs (DCGANs) use convolutional layers to produce high-quality images (17). Conditional GANs (cGANs) can generate medical images with specific diseases, such as tumours or lesions (18). CycleGANs convert images from one domain to another, for example, generating MRI images from CT scan datasets (19). Tabular GANs (TGANs) and Conditional Tabular GANs (CTGANs) are designed to handle numerical and categorical datasets, generating synthetic data conditioned on specific features as specific patient subgroups in certain ages or diagnoses (e.g., patients with diabetes or hypertension). TimeGANs produce time-series data such as ECG (20). Sequence GANs can create synthetic genomic data such as DNA and RNA (21). Variational Autoencoder GANs (VAE-GANs) combine VAE and GANs to generate high-dimensional data as gene expression profiles, for example, creating synthetic data for cancer gene expression analysis. Multimodal GANs generate multiple modalities datasets, such as patient records with associated medical images and reports (22).
  • VAEs are another type of Neural Network used to generate synthetic data. VAEs capture complex data distribution using probabilistic modelling to produce realistic data samples. This neural network learns to encode data into a latent space (learn probability distribution of different data types) and then decode it back to generate synthetic datasets. VAEs generate different data types for medical records, such as image, numerical and bio-signal datasets. VAEs generally have less computational cost than GANs (23) and they do not suffer from the mode collapse issue. Still, it may generate blurrier or less realistic images compared to GAN’s sharp, high-resolution produced images. Conditional VAE (CVAE) can work well with smaller datasets to generate more diverse and representative patient records specifically for rare disease cases. Recent studies demonstrate promising results and high-quality datasets generated by utilising hybrid models like VAE-GANs, which combine the strengths of both VAEs and GANs.

The quality of synthetic data can vary depending on the generation method and the specific use case. No single method is universally superior across all criteria, highlighting the importance of context-specific assessment (24). Additionally, enhancing the features of generated data can help minimise the domain gap between synthetic and real-world data, improving the performance of models trained on synthetic data (25).

Synthetic data can be classified based on the connection to the real data:

• Fully synthetic data: the fabricated data does not have any connection to real data. Created through algorithms without any real observations, this type of generated dataset is utilised when no real data is available or in models required to guarantee confidentiality.
• Partially synthetic data: created datasets can be considered as combination between real data values and fabricated ones. So, some true values remain in the dataset, increasing disclosure risk while maintaining a higher analytical validity.

Accordingly, the two types mentioned, organisations and researchers looking to publish synthetic datasets must determine which method best balances the trade-off between data usefulness and the risk of disclosure.

## Use cases of SD in rare disease research

Synthetic data generation has emerged as a promising solution to overcome the challenges posed by data scarcity and privacy concerns in training AI models for diagnosing rare diseases (26). This approach is particularly valuable in rare genetic disorders, where complex symptoms and lengthy diagnostic processes often hinder timely identification (27). Synthetic data generation not only enhances diagnostic accuracy but also accelerates innovation in the field of rare disease identification and management (28). By replicating the statistical properties of real-world data without exposing sensitive information, synthetic data supports diverse applications, including training AI models, simulating clinical trials, and facilitating cross-institutional collaboration. This section examines various use cases, enriched by insights into privacy and security challenges and solutions in synthetic data methodologies, illustrating its transformative potential in rare disease research.

Within clinical studies of AI, there is a major gap in finding combinations of different modalities, such as genetics, imaging, clinical data for patients. The data for each variable is usually found in its own. For diagnosis, medical experts usually use different elements of evaluation for the patient cases. Synthetic data can generate heterogeneous data types (imaging, clinical data, age, demographics, etc.) to improve AI understanding for rare diseases. The generated data allows simulating hypothetical scenarios or varying conditions with full patient profiles to study disease behaviour, improving diagnostic accuracy or optimising clinical studies, such as patients’ respond to certain compound or side effects. Moreover, genomic data is highly sensitive with privacy laws like GDPR and HIPAA restricting the sharing of real patient data. This necessitates the usage of synthetic data, which can simulate realistic genomic sequences on different demographics such as races and ethnicities. This can effectively assist with having machine models that could discover drug targets and predict the prevalence and effect of rare genetic variants in larger populations (29).

Synthetic data enhances AI model training and validation, especially when actual data is scarce or restricted due to privacy concerns. Generative Adversarial Networks (GANs) are used to create synthetic medical images, such as chest X-rays and brain MRIs, to augment datasets. Combining synthetic and actual data improves classification accuracy, with studies showing 85.9% accuracy in brain MRI classification (30). Synthetic imaging datasets simulate underrepresented clinical scenarios, like non-optimally positioned X-rays, improving model robustness with Dice score enhancements of 3%–15% (31).

Synthetic data efficiently designs and simulates clinical trials, especially in rare disease research. Methods like CTAB-GAN+ and normalising flows (NFlow) create synthetic cohorts replicating demographic, molecular, and clinical characteristics. These datasets significantly enhance studies on Acute Myeloid Leukaemia (AML), capturing survival curves and complex inter-variable relationships (32). By reducing research time and costs, synthetic data accelerates clinical advancements. D’Amico et al. (33) report a threefold increase in a synthetic cohort based on 944 myelodysplastic syndrome (MDS) patients, predicting molecular classification results years before real-world data collection.

Privacy-preserving synthetic data also enables secure cross-institutional research while minimising data breach risks. According to (34), multiple parties can generate privacy-preserving synthetic datasets using differentially private generative modelling techniques. However, challenges such as data breaches and model inversion attacks remain a concern (35). Differentially private GANs mitigate these risks, enabling institutions to securely combine datasets for improved analytical accuracy and reduced bias (34). Collaborative frameworks using synthetic data address key vulnerabilities, such as insufficient anonymisation and weak access controls (36). By removing identifiable information, synthetic data ensures GDPR and HIPAA compliance and supports robust cross-border collaborations.

The application of synthetic data in rare disease research offers promising solutions to challenges in diagnostics, clinical trials, and collaborative studies. By enabling secure data sharing, improving AI model training, and accelerating research timelines, synthetic data provides a path forward for innovation in rare disease management. However, addressing emerging privacy and security challenges, as highlighted in the broader context of federated learning and data breach prevention, remains essential to ensure trust and reliability in synthetic data solutions. As synthetic data techniques evolve, they will further expand their impact on rare disease research and healthcare innovation.

## How synthetic data overcomes key challenges in rare disease research

Research in medical artificial intelligence-based studies is often constrained by the availability of datasets, for privacy reasons, or the rarity of certain diseases or conditions. Developing new treatments using machine learning techniques or evaluating the treatment efficacy can be seen as data-driven approaches. These data driven approaches including deep learning algorithms, require large datasets for model validation and training. Synthetic data presents an innovative solution to the challenge of data scarcity in medical research. By producing artificial datasets that mirror real-world statistical patterns, synthetic data serves as a necessary resource for researchers, enabling them to overcome data constraints and further their research objectives (6).

First, rare disease can be defined as a disease affecting small number of people. This poses a challenge for AI researchers, as the limited data available is insufficient for building robust models to develop treatments. Synthetic datasets allow researchers to study cases with larger amounts of information distributed across diverse patient demographics, including age, gender, race, ethnicity. This eliminates the need to wait for real clinical data from multiple countries and institutions to become available.

Second, the lack of patient case information presents another challenge. Medical datasets are often restricted due to data privacy and ethical concerns, limiting their availability. However synthetic data do not contain any sensitive health information or real personal data. Ethically, such fabricated datasets can be freely shared, stored and utilised in analysing rare disease without strict privacy regulations.

Third, synthetic patient data will achieve the generalisability of AI models. Rare diseases often involve genetic variations, environmental factors, medical histories, and treatment scenarios, making it difficult to capture a comprehensive range of cases using real-world data alone. By providing a large and diverse dataset, synthetic data helps improve the accuracy of the AI models, allowing medical research to overcome the time constraints and limitations associated with real datasets.

## Case studies

Generating datasets for rare diseases proposes a valuable asset for researches providing important insights into disease diagnosis, drug discovery, and treatment effectiveness accross diverse populations with varying factors. The generated data include medical images, statistical data, and natural language processing data. They may consist of synthetic patient records (uch as age, sex, and ethnicity), medical history, clinical data, symptoms, treatment response, and genomic information. Another category is the synthetic medical images such as X-rays and MRI.

Yelmen et al. (37) shows that deep generative adversarial networks (GANs) and restricted Boltzmann machines (RBMs) can generate high-quality artificial genomes datasets (37). They mimic features of real genomic datasets, considering the generated Artificial Genomes (AG) as a valuable asset for genetic studies, particularly for underrepresented populations. The experiments used genomic data from 2,504 individual genomes from 1,000 Genomes Project and 1,000 individuals from Estonian Biobank to create artificial genomes (AGs). Additionally, to different datasets were used for imputating low frequency alleles and testing. The experimental results proved that both models effectively encoded the data, with RBMs performing better in capturing rare genetic variations.

The research highlights a major drawback of the proposed models: no fully artificial whole genomes have been produced, due to computational limitations. Instead, only devise genomic snippets have been generated. Another reported issue is that half of the rare alleles remains fixed in the GANs AGs, while RBMs AGs better capture the rare alleles present in real genomes.

European Health Data & Evidence Network (EHDEN) (38) provides the required infrastructure for the healthcare data analytics field (38). EHDEN collaborates on a federated network across Europe with different researchers, offering innovative solutions using synthetic data to address the challenges of rare disease research gap, accelerating researches, ensure patients data privacy, and standardize health data to common data model (CDM) (39).

Voss et al. (40) research proves that the workflow used by EHDEN effectively supports the successful standardisation of observational data across Europe. Their study involved 25 data partners from 11 countries who received funding from the EHDEN to standardise their data. The results were measured by days required to convert health observations to Observational Medical Outcomes Partnership, OMOP CDM.

Krastev (41) presents an application to convert more than 380 million pseudonymised outpatient records to OMOP CDM to be published in EHDEN and used on large scale studies. The study focusses on the need for preprocessing the data structure of raw data, cleaning data and procedures for assuring data quality (41).

Al-Dhamari (42) propose a software tool called SynthMD, a lightweight librarydesigned to generate sunthetic datasets following the set of collected statistical distributions from US such as different race, age and gender, clinical data, and survive rate. The experiments focused on three specific rare diseases—Sickle Cell Disease, Cystic Fibrosis, and Duchenne Muscular Dystrophy—each with specific concerns related to gender and racial groups. However, the study’s main limitation was that it generated only tabular data and captured a limited range of statistical information.

Oliveira et al. (43) compare ten different GAN architectures for generating synthetic eye-fundus images, both with and without Age-related Macular Degeneration (AMD). The study combined data from three public databases (iChallenge-AMD, ODIR-2019, and RIADD) to form a single training and test set. Researchers claim that even clinicians struggled to distinguish between real and synthetic images. Experiment resulst showed that the ResNet-18 architecture achieved the highest performance with 85% accuracy, outperforming the two human experts (80%, 75%) in detecting AMD fundus images.

## The role of SD in addressing regulatory and ethical concerns

Synthetic data address healthcare privacy, regulatory, and ethical concerns in healthcare by enhancing data privacy, improving predictive analytics, and informing policy decisions (6). It helps mitigate the risks associated with using authentic biometric data for AI training, particularly face recognition technology (44). Additionally, synthetic data support compliance with privacy regulations such as GDPR and HIPAA while by replicating the statistical properties of actual patient data without including personal information. This aligs with data minimisation and purpose limitation principles (45, 46).

However, synthetic data must be carefully generated to avoid reidentification risks and conflicts with GDPR’s protection of individual rights and freedoms (47). Ethically, synthetic data offers advantages by reducing the potential for data misuse, patient reidentification, and consent-related issues. Privacy-preserving simulations enable researchers to analyse sensitive data while yielding similar results to original data (6, 48, 49).

Synthetic data can also replicate complex real-world data, including those related to rare disease. However, achieving complete realism remains a challenge, as synthetic data may not fully capture all nuances necessary for high research value. Despite advancements in generating realistic biomedical datasets, such as fully synthetic EHRs, that facilitate data sharing and methodological research (50), limitations persist. Frameworks like stdpopsim enable realistic genome-scale simulations across various species, including non-model organisms (51). However, replicating the complexity of rare disease data remains remains difficult. This challenge is evident in fields like image-deraining research, where models trained on synthetic data underperform on real-world scenarios (52). Some educational contexts (53) may provide effective approaches for generating indicating more realistic and valuable synthetic data for rare disease research. The limitations suggest that combining synthetic data and real-world data may be necessary to enhance to enhance research validity and clinical applicability.

## Current limitations and challenges

While synthetic data generation offers significant benefits, synthetic datasets face several limitations that must be addressed to ensure their effective use in rare disease research. Key challenges include maintaining data quality, mitigating bias, validating datasets, and overcoming computational resource demands.

Bias in real-world datasets can be propagated or even amplified during synthetic data generation, which is particularly concerning when the source data underrepresents specific populations or medical conditions, leading to unreliable AI model performance. Mitigating these biases may involve re-sampling, re-weighting, and adversarial debiasing techniques that can be employed during data generation (54). Implementing fairness-aware algorithms and auditing synthetic datasets with third-party reviews can also ensure a balanced representation across demographic and clinical subgroups (24). Evaluating the synthetic dataset against fairness metrics such as demographic parity, equal opportunity, and disparate impact can help identify and correct biases.

The validation of synthetic datasets is critical to ensuring their utility and comparability to real-world data. Metrics for evaluating the quality of synthetic data can include statistical similarity measures, such as Wasserstein distance, Kullback-Leibler divergence, and Jensen-Shannon divergence, which assess how closely the synthetic data matches the statistical distributions of the actual data (55). Utility metrics, such as predictive accuracy, precision, recall, and F1-score, can measure how well AI models trained on synthetic data perform real-world tasks (56). Domain expert assessments are also vital for validating whether the synthetic data aligns with real-world medical knowledge and expectations.

Generating high-quality synthetic data is computationally intensive, particularly for complex data types like medical imaging or high-dimensional clinical datasets. Training advanced generative models requires significant computational power and time (57). For example, GANs require a large amount of data to train the generator efficiently. These challenges can be mitigated using certain methods to handle the computational overhead issue of GANs. Methods such as Transfer Learning (TL), using pre-trained GAN models and only fine-tuning them on smaller datasets to adapt the process to the required task, or hybrid models which depend on fusion of different generative models, are two possible methods to handle GANs complexity and computational burden. Another alternative is leveraging cloud-based platforms offering scalable computing resources, optimising model architectures to reduce computational overhead, and adopting federated learning frameworks to distribute workloads across multiple nodes (58).

Generating high-quality synthesised datasets for rare diseases encounters data scarcity, high dimensionality and privacy concerns (59). Also, it requires addressing issues of bias, ensuring robust validation, and overcoming computational challenges to provide robust information that empowers meaningful insights into rare diseases.

Researchers can use fairness-aware algorithms, rigorous validation metrics, and scalable computational strategies to develop reliable synthetic datasets that enhance rare disease research while maintaining data integrity and privacy compliance. These improvements will facilitate the broader adoption of synthetic data in medical research and its integration into real-world applications.

## Future perspectives

Synthetic data represent an unparalleled opportunity to accelerate rare disease research, while safeguarding patient privacy. By addressing critical limitations and fostering innovation, synthetic data can become the cornerstone of future healthcare, enabling equitable, efficient, and collaborative advancements. It holds the potential to transform rare disease research by overcoming barriers such as data scarcity, privacy concerns, and regulatory constraints, which traditionally impede progress.

One of its most promising applications is personalised medicine. By simulating diverse patient profiles, synthetic data enhances AI models’ ability to detect rare diseases across varying demographics and genetic contexts (27, 30). Integrating synthetic data with real-world evidence is essential for refining AI-driven diagnostics and therapeutic strategies, particularly during early clinical validation phases.

Synthetic data also facilitate cross-border research collaborations by adhering to privacy regulations, such as GDPR and HIPAA, enabling international studies without compromising patient confidentiality (26). Federated learning frameworks augmented by synthetic data offer a promising avenue for securely integrating datasets, accelerating global research initiatives while maintaining privacy standards (34).

Emerging technologies such as Generative Adversarial Networks (GANs) and Variational Autoencoders (VAEs) should be leveraged to improve the realism and representativeness of synthetic datasets (30). Hybrid models combining synthetic and real-world datasets provide a robust foundation for developing generalisable AI systems for rare diseases, ensuring inclusivity and accuracy.

However, the ethical considerations are critical. The generation and use of synthetic data requires transparency in methodologies and adherence to de-identification standards to mitigate re-identification risks (36). Collaboration between policymakers, industry leaders, and researchers is essential for establishing unified frameworks for synthetic data usage, ensuring compliance with evolving data governance laws, and fostering public trust.

Validation of synthetic datasets against real-world data is imperative to confirm their clinical and research utility (31). Involving domain experts in this process bridges gaps in realism and analytical value, ensuring that the datasets meet the rigorous standards required for medical research.

Validation of synthetic datasets against real-world data is imperative to confirm their clinical and research utility (31). Involving domain experts in this process bridges gaps in realism and analytical value, ensuring that the datasets meet the rigorous standards required for medical research.

Despite its promise, generating high-quality synthetic data remains computationally demanding and requires specialised expertise. Investment in infrastructure and training is necessary to overcome these barriers. Research on ethical frameworks and international standards must also be prioritised to ensure long-term viability and stakeholder trust.

Unified standards for generating, sharing, and validating synthetic data, combined with pilot programmes within frameworks such as the European Health Data Space (EHDS), can streamline research efforts and foster innovation, driving transformative progress in healthcare research.

## Conclusion

Synthetic data have emerged as a transformative tool in rare disease research, offering innovative solutions to overcome the challenges of data scarcity, privacy concerns, and regulatory constraints. By enabling the generation of realistic, yet non-identifiable datasets, synthetic data facilitate the training of AI models, simulation of clinical trials, and cross-border collaborations, while maintaining compliance with privacy regulations such as GDPR and HIPAA. These advancements have significantly enhanced the capabilities of researchers to diagnose, treat, and manage rare diseases more effectively.

The integration of synthetic data with real-world evidence has demonstrated the potential to refine AI-driven diagnostic tools and accelerate therapeutic development. Case studies, such as those employing Generative Adversarial Networks (GANs) to generate synthetic medical imaging or CTAB-GAN+ models for rare disease clinical trials, have highlighted their ability to mimic complex datasets with high fidelity. These applications not only optimise research timelines but also reduce the resource demands associated with traditional methods.

Despite their promise, synthetic data generation still faces challenges, including the need for advanced computational resources, rigorous validation, and ethical governance. Addressing biases, ensuring data representativeness, and fostering public trust using transparent methodologies remain critical. Collaborative efforts between policymakers, researchers, and industry stakeholders are essential for establishing unified standards and ethical frameworks for their use.

By bridging data gaps and fostering global collaboration, synthetic data have the potential to revolutionise healthcare research. However, realising its full potential requires continued innovation, investment, and adherence to rigorous ethical and regulatory standards to ensure sustainable progress in rare disease research.

## References

[1] Boulanger V Schlemmer M Rossov S Seebald A Gavin P. Establishing patient registries for rare diseases: rationale and challenges. Pharmaceut Med. (2020) 34:185–90. 10.1007/s40290-020-00332-1 32215853 PMC7286934

[2] Devuyst O Meij I Antignac PMDC Levtchenko EN Muller D Hoff WGV Eunefron, the european network for the study of orphan nephropathies. Nephrol Dial Transplant. (2009) 24:2011–5. 10.1093/ndt/gfp095 19264741

[3] Facey K Wong-Rieger D Shah N Kent A Wilt GJVD. Generating health technology assessment evidence for rare diseases. Int J Technol Assess Health Care. (2014) 30:416–22. 10.1017/s0266462314000464 25407328

[4] Mcglinn K Rutherford MA Gisslander K Hederman L Little MA O’Sullivan D. Fairvasc: a semantic web approach to rare disease registry integration. Comput Biol Med. (2022) 145:105313. 10.1016/j.compbiomed.2022.105313 35405400

[5] Mascalzoni D Paradiso A Hansson M. Rare disease research: breaking the privacy barrier. Appl Transl Genomics. (2014) 3:23–9. 10.1016/j.atg.2014.04.003 PMC4882025 27275410

[6] Giuffré M Shung DL. Harnessing the power of synthetic data in healthcare: innovation, application, and privacy. npj Digit Med. (2023) 6:186. 10.1038/s41746-023-00927-3 37813960 PMC10562365

[7] Jadon A Kumar S. Leveraging generative AI models for synthetic data generation in healthcare: balancing research and privacy. In: 2023 International Conference on Smart Applications, Communications and Networking (SmartNets). (2023). p. 1–4.

[8] Aziz M Khan AJ Khan S. Rare disease registries – purpose, challenges & solutions. Int J Adv Res. (2021) 4:1190–6. 10.21474/IJAR01/12392

[9] Ibrahim N. Navigating the complexity of rare diseases: challenges, innovations, and future directions. Glob J Med Ther. (2023) 5:12–22. 10.46982/gjmt.2023.108

[10] Jia J Shi T. Towards efficiency in rare disease research: what is distinctive and important? Sci China Life Sci. (2017) 60:686–91. 10.1007/s11427-017-9099-3 28639105

[11] Kole A Faurisson F. Rare Diseases Social Epidemiology: Analysis of Inequalities. Dordrecht: Springer Netherlands (2010). p. 223–50. 10.1007/978-90-481-9485-8_14 20824449

[12] Fonseca DA Amaral I Pinto AC Cotrim MD. Orphan drugs: major development challenges at the clinical stage. Drug Discov Today. (2019) 24:867–72. 10.1016/j.drudis.2019.01.005 30658132

[13] Ahmed MA Krishna R Kartha RV Shang E Abuasal B Bakhaidar R Getting the dose right in drug development for rare diseases: barriers and enablers. Clin Pharmacol Ther. (2024) 16:1412–32. 10.1002/cpt.3407 39148459

[14] Smith CT Beresford MW Williamson PR. Methodology of clinical trials for rare diseases. Best Pract Res Clin Rheumatol. (2014) 28:247–62. 10.1016/j.berh.2014.03.004 24974061

[15] Sriram P. Beyond placebo: alternative options to the randomized control trial design in rare disease studies. Clin Trials Pract Open J. (2020) 3:1–4. 10.17140/ctpoj-3-110

[16] Irmak DK. Orphan drugs: getting arms around rare diseases. J Community Public Health Nurs. (2017) 3:2. 10.4172/2471-9846.1000167

[17] Skandarani Y Jodoin PM Lalande A. GANs for medical image synthesis: an empirical study. J Imaging. (2023) 9:69. 10.3390/jimaging9030069 36976120 PMC10055771

[18] Raad R Ray D Varghese B Hwang D Gill I Duddalwar V Conditional generative learning for medical image imputation. Sci Rep. (2024) 14:171. 10.1038/s41598-023-50566-7 38167932 PMC10762085

[19] Czobit C Samavi R. Cyclegan models for MRI image translation. *CoRR* [Preprint]. abs/2401.00023 (2024).

[20] Huang F Deng Y. Tcgan: convolutional generative adversarial network for time series classification and clustering. Neural Netw. (2023) 165:868–83. 10.1016/j.neunet.2023.06.033 37433231

[21] Berman DS Howser C Mehoke T Ernlund AW Evans JD. Mutagan: a sequence-to-sequence gan framework to predict mutations of evolving protein populations. Virus Evol. (2023) 9:vead022. 10.1093/ve/vead022 37066021 PMC10104372

[22] Ziegler JD Subramaniam S Azzarito M Doyle O Krusche P Coroller T. Multi-modal conditional GAN: data synthesis in the medical domain. In: NeurIPS 2022 Workshop on Synthetic Data for Empowering ML Research (2022).

[23] Sahoo R Naik V Singh S Malik S. Gans and vaes as methods of synthetic data generation and augmentation to enhance heart disease prediction. Int J Eng Adv Technol. (2021) 11:17–23. 10.35940/ijeat.B3263.1211221

[24] Yan C Yan Y Wan Z Zhang Z Omberg L Guinney J A multifaceted benchmarking of synthetic electronic health record generation models. Nat Commun. (2022) 13:7609. 10.1038/s41467-022-35295-1 36494374 PMC9734113

[25] Aranjuelo N García S Loyo E Unzueta L Otaegui O. Key strategies for synthetic data generation for training intelligent systems based on people detection from omnidirectional cameras. Comput Electr Eng. (2021) 92:107105. 10.1016/j.compeleceng.2021.107105

[26] Pezoulas VC Zaridis DI Mylona E Androutsos C Apostolidis K Tachos NS Synthetic data generation methods in healthcare: a review on open-source tools and methods. Comput Struct Biotechnol J. (2024) 23:2892–910. 10.1016/j.csbj.2024.07.005 39108677 PMC11301073

[27] Liv B. Advanced AI and augmented reality (AR) integration in medical and surgical practice. Next Front Life Sci AI. (2024) 8:35. 10.62802/x9ae7523

[28] Kumar A. The role of synthetic data in advancing ai models: opportunities, challenges, and ethical considerations. J Artif Intell Gen Sci. (2024) 5:443–59. 10.60087/jaigs.v5i1.256

[29] Achuthan S Chatterjee R Kotnala S Mohanty A Bhattacharya S Salgia R Leveraging deep learning algorithms for synthetic data generation to design and analyze biological networks. J Biosci. (2022) 47:43. 10.1007/s12038-022-00278-3 36222162

[30] Dhawan K Nijhawan SS. Cross-modality synthetic data augmentation using GANs: enhancing brain MRI and chest x-ray classification. medRxiv [Preprint]. (2024). 10.1101/2024.06.09.24308649

[31] Fok WYR Kappler S Saalfeld S Geiger B Biniazan R Fieselmann A Adversarial robustness improvement for x-ray bone segmentation using synthetic data created from computed tomography scans. Springer Sci Bus Media LLC. (2024) 14:25813. 10.21203/rs.3.rs-4473429/v1 PMC11519576 39468116

[32] Eckardt J-N Schetelig J Serve H Sedlmayr M Wolfien M Middeke JM Mimicking clinical trials with synthetic acute myeloid leukemia patients using generative artificial intelligence. npj Digit Med. (2024) 7:76. 10.1038/s41746-024-01076-x 38509224 PMC10954666

[33] D’Amico S Castellani G Sala C Dall’Olio D Bicchieri M Tentori C Synthetic data generation by artificial intelligence to accelerate research and precision medicine in hematology. JCO Clin Cancer Inform. (2023) 7:e2300021. 10.1200/cci.23.00021 37390377 PMC10569771

[34] Prediger L Jälkö J Honkela A Kaski S. Collaborative learning from distributed data with differentially private synthetic data. BMC Med Inform Decis Mak. (2024) 24:167. 10.1186/s12911-024-02563-7 38877563 PMC11179391

[35] Zhang J Xu Q Wang F Zhu H Zhao J Li H. Security and privacy threats to federated learning: issues, methods, and challenges. Secur Commun Netw. (2022) 2022:1–24. 10.1155/2022/2886795

[36] Schlackl F Link N Hoehle H. Antecedents and consequences of data breaches: a systematic review. Inform Manage. (2022) 59:103638. 10.1016/j.im.2022.103638

[37] Yelmen B Decelle A Ongaro L Marnetto D Tallec C Montinaro F Creating artificial human genomes using generative neural networks. PLoS Genet. (2021) 17:e1009303. 10.1371/journal.pgen.1009303 33539374 PMC7861435

[38] EHDEN. Data from: European health data & evidence network: Advancing health research with synthetic data solutions in a gdpr-compliant framework. EHDEN Project Documentation (2022).

[39] CDM. Data from: Omop common data model. Available online at: https://github.com/OHDSI/ (accessed November 22, 2024).

[40] Voss EA Blacketer C van Sandijk V Moinat M Kallfelz M van Speybroeck M European health data & evidence network–learnings from building out a standardized international health data network. J Am Med Inform Assoc. (2023) 31:209–19. 10.1093/jamia/ocad214 37952118 PMC10746315

[41] Krastev E Markov E Abanos S Krasteva R Tcharaktchiev D. Mapping the bulgarian diabetes register to OMOP CDM: Application results. Stud Health Technol Inform. (2024) 313:28–33. 10.3233/SHTI240007 38682500

[42] Al-Dhamari I Attieh A Prasser F. Synthetic datasets for open software development in rare disease research. Orphanet J Rare Dis. (2024) 19:265. 10.1186/s13023-024-03254-2 39010138 PMC11247768

[43] Oliveira GC Rosa GH Pedronette DC Papa JP Kumar H Passos LA Robust deep learning for eye fundus images: bridging real and synthetic data for enhancing generalization. Biomed Signal Process Control. (2024) 94:106263. 10.1016/j.bspc.2024.106263

[44] Boutros F Struc V Fierrez J Damer N. Synthetic data for face recognition: current state and future prospects. Image Vis Comput. (2023) 135:104688. 10.1016/j.imavis.2023.104688

[45] Caruccio L Desiato D Polese G Tortora G. Gdpr compliant information confidentiality preservation in big data processing. IEEE Access. (2020) 8:205034–50. 10.1109/access.2020.3036916

[46] Crutzen R Peters G-JY Mondschein C. Why and how we should care about the general data protection regulation. Psychol Health. (2019) 34:1347–57. 10.1080/08870446.2019.1606222 31111730

[47] Bertolaccini L Batirel H Brunelli A Passani S Szanto Z Falcoz P-E The significance of general data protection regulation in the compliant data contribution to the European society of thoracic surgeons database. Eur J Cardiothorac Surg. (2023) 64:ezad289. 10.1093/ejcts/ezad289 37589648

[48] Husedzinovic A Winkler EC FrÖhling S Ose D Schickhardt C. Stakeholders’ perspectives on biobank-based genomic research: systematic review of the literature. Eur J Hum Genet. (2015) 23:1607–14. 10.1038/ejhg.2015.27 25735479 PMC4795193

[49] Guillaudeux M Limou S Vince N Karakachoff M Wargny M Rousseau O Patient-centric synthetic data generation, no reason to risk reidentification in biomedical data analysis. Npj Digit Med. (2023) 6:37. 10.1038/s41746-023-00771-5 36899082 PMC10006164

[50] Lee SH. Natural language generation for electronic health records. Npj Digit Med. (2018) 1:63. 10.1038/s41746-018-0070-0 30687797 PMC6345174

[51] Lauterbur M Adrion J Cury J Pope N Ragsdale A Iasi L Expanding the stdpopsim species catalog, and lessons learned for realistic genome simulations. eLife. (2023) 12:RP84874. 10.7554/elife.84874 37342968 PMC10328510

[52] Huang H He R Luo M. Memory uncertainty learning for real-world single image deraining. IEEE Trans Pattern Anal Mach Intell. (2023) 45:3446–60. 10.1109/tpami.2022.3180560 35671310

[53] Singer JD Willett JB. Improving the teaching of applied statistics: putting the data back into data analysis. Am Stat. (1990) 44:223–30. 10.1080/00031305.1990.10475726

[54] Chundawat VS Narang P Mandal M Lahoti M Tarun AK. A universal metric for robust evaluation of synthetic tabular data. IEEE Trans Artif Intell. (2024) 5:300–9. 10.1109/tai.2022.3229289

[55] Torfi A Fox EA Reddy CK. Differentially private synthetic medical data generation using convolutional GANs. Inf Sci. (2021) 586:485–500. 10.1016/j.ins.2021.12.018

[56] Rajotte J-F Bergen R Buckeridge DL Emam KE Ng R Strome E. Synthetic data as an enabler for machine learning applications in medicine. IScience. (2022) 25:105331. 10.1016/j.isci.2022.105331 36325058 PMC9619172

[57] Eno J Thompson CW. Generating synthetic data to match data mining patterns. IEEE Internet Comput. (2008) 12:78–82. 10.1109/mic.2008.55

[58] Dahmen J Cook D. Synsys: a synthetic data generation system for healthcare applications. Sensors. (2019) 19:1181. 10.3390/s19051181 30857130 PMC6427177

[59] Gonzales A Guruswamy G Smith SR. Synthetic data in health care: a narrative review. PLoS Digit Health. (2023) 2:e0000082. 10.1371/journal.pdig.0000082 36812604 PMC9931305

